# Implementation of ddaE neuron growth mechanism in graph grammar replicates biological features

**DOI:** 10.64898/2026.07.27.741078

**Published:** 2026-07-30

**Authors:** Matthew Hur, Patrick T. Hwu, Katherine L. Thompson-Peer, Eric D. Mjolsness

## Abstract

Dendrites develop branching patterns that are critical for their function, yet the mechanisms guiding arbor morphology remain incompletely understood, and quantitative models predicting how signals guide morphology remain limited. The ddaE neuron in *Drosophila* larvae is a proprioceptive sensory neuron with a characteristic asymmetric dendrite arbor that exhibits posterior-biased branching. We developed a computational model using Dynamical Graph Grammar (DGG) to simulate ddaE dendrite development as a graph-based dynamical system, using a single morphogen gradient to establish arbor architecture. Our simulations of ddaE dendrites, guided by the spatial gradient of the Teneurin-m (Tenm) morphogen combined with resource constraints and self-avoidance rules, accurately recapitulate the morphological features of biological ddaE neurons, including primary branch orientation, posterior bias, branch tree distributions, and branch length statistics. We find that the response to a single morphogen gradient is sufficient to guide the computerized dendritic arbor. Null model analyses demonstrate that simulated arbors exhibit non-random spatial and topological organization consistent with biological constraints. Our results demonstrate that rules based on a single morphogen gradient are sufficient to generate complex asymmetric dendritic patterns and provide a validated computational framework for testing perturbations *in silico* .

**SIGNIFICANCE:**

We develop and simulate a minimal computational model of dendritic arbor morphogenesis based on a single morphogen gradient. Asymmetric dendrite arbors, such as the ddaE proprioceptive neuron in Drosophila larvae, have not previously been computationally modeled. Using the Dynamical Graph Grammar framework, we create a mathematical model from 17 dynamical rules governing changes in arbor structure, spatial position, morphogen-directed growth, and the local dynamic state of each tip. Each tip switches among three states: growth/pause/shrinkage, while tips that encounter another branch additionally enter a retraction state. Using a large simulation sample size, we characterize our model system’s generative outputs and verify that they match imaged biological dendrites across the majority of morphological statistics, including posterior branch bias, branch degree distributions, branch number, and dendrite length. We demonstrate that efficient spacing is guided by the orientation of branch junctions. We make our simulation publicly available to function with high-throughput investigation of gene-to-phenotype relationships in dendrite development.

## Full Text Availability

The license terms selected by the author(s) for this preprint version do not permit archiving in PMC. The full text is available from the preprint server.

